# Endoscopically Created Dual-Path Intestinal Diversion Using an Incision-Less Anastomosis System in Obese Subjects: 3-Year Results of Nutrition Observation

**DOI:** 10.33549/physiolres.935757

**Published:** 2025-12-01

**Authors:** Marek BUŽGA, Evžen MACHYTKA, Zdeněk ŠVAGERA, Jitka MACHÁČKOVÁ, Karolína JANOCHOVÁ, Vladislava MIZEROVÁ

**Affiliations:** 1 Institute of Laboratory Diagnostics, University Hospital of Ostrava Faculty of Medicine, Ostrava, Czech Republic; 2 Department of Physiology and Pathophysiology, Medical faculty, University of Ostrava, Czech Republic; 3 Department of Gastroenterology, University Hospital of Ostrava, Ostrava, Czech Republic; 4 Institute of Laboratory Medicine, Faculty of Medicine, University of Ostrava, Ostrava, Czech Republic

**Keywords:** Anastomosis, Endoscopy, Magnetic, Obesity, Nutrition deficiencies

## Abstract

Obesity is associated with multiple metabolic disturbances and nutritional risks. Minimally invasive small-intestine interventions are being developed as alternatives to bariatric surgery, aiming to induce weight reduction with a lower risk of malabsorption. This 3-year pilot study evaluated the safety, metabolic effects, and nutritional outcomes of a novel incisionless magnetic anastomosis system (IMAS) creating an endoscopic dual-path intestinal diversion in obese subjects. Ten obese adults (mean BMI 41 kg/m^2^) underwent endoscopic creation of a jejuno-ileal diversion using the IMAS device. The cohort included subjects with normal glycemia, prediabetes, and type 2 diabetes. Clinical and biochemical parameters were monitored at baseline and at 6-, 12-, 18-, 24-, and 36-month visits. Anthropometry and a full micronutrient profile (vitamins A, E, B1–B12, D, folate, ferritin, albumin, total protein) were assessed. Mean body weight decreased from 120.9±17.8 kg to 101.9±22.2 kg after 36 months (p<0.05), with an excess weight loss of 43.2 %. Serum concentrations of most micronutrients remained within physiological limits; only vitamin B12 and 25(OH)D showed significant declines during long-term follow-up. The procedure was generally well tolerated, with mild gastrointestinal symptoms resolving over time. Endoscopically created dual-path intestinal diversion achieved durable weight reduction over 3 years with a favorable nutritional risk profile. The incidence of micronutrient deficiencies was substantially lower than that reported after malabsorptive bariatric surgery. These results support the potential of this incisionless technique as a less invasive option for obesity management and warrant larger controlled trials.

## Introduction

Obesity and its metabolic complications remain a major global health burden. While bariatric surgery achieves durable weight loss, its uptake is limited by invasiveness and long-term nutritional concerns [1]. These limitations have accelerated development of bariatric and metabolic endoscopy, including small-intestine-targeted interventions [2].

Among these, partial jejunal diversion (PJD) created by an incisionless magnetic anastomosis system (IMAS) establishes a dual-path intestinal flow while preserving the native lumen [3]. In 2017, our group published the first-in-human pilot of PJD using IMAS [4]; ten patients with obesity and type 2 diabetes mellitus, prediabetes, or no diabetes were enrolled and followed for one year. The present manuscript reports 36-month follow-up of that original cohort, focusing on nutritional outcomes. The concept was first reported by Ryou *et al*., who constructed a gastrojejunostomy and subsequently a dual path anastomosis in porcine models [5]. The anastomosis allows a portion of ingested nutrients to circumvent a majority of the small bowel. Because the native path remains open, this dual-path intestinal diversion is not a jejuno-ileal bypass (i.e., not a blind or defunctionalized segment of small intestine, which can result in a number of serious adverse events [AEs] due to malabsorption).

Concurrently, other small-intestine–directed endoscopic approaches – most notably the duodenal-jejunal bypass liner (DJBL) – show clinically meaningful 12-month reductions in BMI and HbA1c, though device removal often limits long-term nutritional assessment [6,7]. Stomach-targeted endoluminal procedures such as endoscopic sleeve gastroplasty (ESG) generally report a low prevalence of significant micronutrient deficiencies in the first postoperative year, differing from malabsorptive surgery risk profiles [8].

Long-term surgical literature consistently documents vitamin and mineral deficiencies (e.g., vitamin D, B12, iron) years after bypass procedures, underscoring the need for structured surveillance [1,9]. Whether similar patterns emerge after permanent endoscopic dual-path diversion is unclear. By prioritizing nutritional status at 36 months – including body composition and a comprehensive micronutrient panel with a full vitamin profile – this study aims to fill a critical evidence gap and complement existing evidence on surgical and temporary endoscopic interventions [10].

Aim of this study was to conduct long-term monitoring of obese patients undergoing PJD and the assess the effect of PJD on the nutritional status of these patients and the biochemical blood parameters.

## Methods

The study was approved by the Ethics Committee at the Faculty of Medicine, University of Ostrava, in accordance with the ethical standards of the Helsinki Declaration of 1975, as amended in 2000. The study had a prospective, observational, and open-label design (ClinicalTrials.gov registration: NCT02839512). Inclusion criteria included a BMI 35–40 kg/m^2^ and an age of 21–64 years, as per IFSO criteria. Exclusion criteria included a BMI>40 kg/m^2^, GI stenosis or obstruction, previous bariatric surgery, balloon or other endoscopic obesity-related therapy, gastro-duodenal ulcer and absorption disorders.

### Endoscopic procedure

**Fig. 1:**
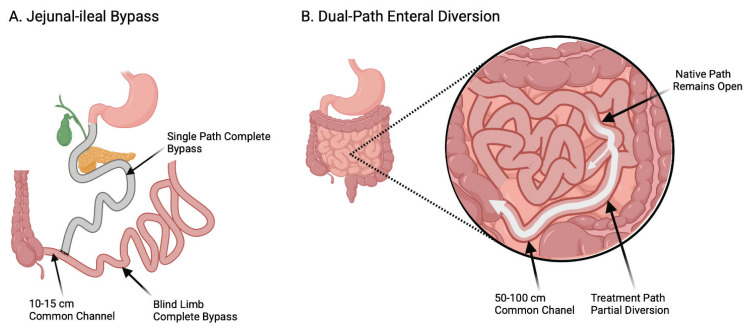
Jejunal-ileal bypass vs. dual-pathway diversion (Created in BioRender. Bužga, M. (2025) https://BioRender.com/y12t681)

IMAS was implanted to create jejuno-ileal intestinal diversion in individuals who met the entry criteria. The magnets of the IMAS system were introduced simultaneously with a colonoscope and an enteroscope. The exact methodology of the procedure has already been published in Machytka *et al*. [18]. This method is illustrated in comparison with jejunal-ileal bypass in Figure 1.

### Anthropometric parameters

The patients’ body weight and height were measured at each visit (before surgery and then 6, 12, 18, 24, and 36 months after surgery). The height was measured in centimeters. The weight was measured in kilograms by a weighing machine with a calibration up to a weight of 250 kg.

### Blood tests

Blood draws were performed the morning after an overnight fast, prior to the scheduled procedure, and subsequently at visits 6, 12, 18, 24, and 36 months. Blood samples were processed for analysis within 20 min of collection. Serum concentrations of glucose, prealbumin, albumin, total protein and iron were assessed (AU 5820, Beckman Coulter, Inc., Brea, CA, USA). Analysis of all parameters showed inter-assay variation coefficients lower than 5 %.

Insulin, folic acid, and vitamin B12 were determined by Access DxI (Beckman Coulter, Inc., Brea, CA, USA) with inter-assay CV 4.1, 4.8, 6.1, 6.4 %. Ferritin was determined by Centaur (Siemens Healthcare Diagnostics Inc., Tarrytown, NY, USA) with CV 4.7, 4.6 %. HbA1c was assessed by Tosoh G8 (Tosoh Corporation, Tokyo, Japan) with inter-assay CV 1.6 %. To reduce analytical variation, vitamins derived from fat tissue from all patients were analyzed in the same run (i.e. simultaneously). Blood samples were stored at −80 °C until analysis. Vitamin A, vitamin E, vitamin C, 25(OH)D in plasma were performed on Agilent 1100 HPLC system with UV/VIS detection (Agilent Technologies, Inc., CA, USA) using RECIPE kits (RECIPE, Munchen, Germany) with inter-assay CV lower than 5 %. Vitamin B1, B2 in whole blood and vitamin B6 in plasma were measured by Hitachi Lachrom HPLC system with FLD (Hitachi High-Technologies Corporation, Tokyo, Japan) using RECIPE kits (RECIPE, Munchen, Germany) with inter-assay CV lower than 5 %.

### Statistical analysis

For all measured parameters, the degree of position (mean) and the degree of variability (standard deviation) were characterized. To verify the normality of the data distribution, we performed a Shapiro-Wilk test. A parametric independent *t*-test is used to assess the statistical significance of the differences in means. The level of statistical significance was chosen for all tests used at the level α=0.05. A paired *t*-test (for continuous variables) or Wilcoxon’s test (for categorical data) was computed to compare follow-up outcomes with baseline values to aid in interpretation. For fasting glycemia (mmol/l) and HbA1c we summarize data as median [IQR] together with 95 % confidence intervals for the median at each time point, stratified by clinical subgroups (diabetics, prediabetics, non-diabetics). The 95 % CIs for the median were computed using a nonparametric exact method based on binomial order statistics (sign-test approach). HbA1c values were standardized to IFCC units (mmol/mol); entries plausibly recorded in NGSP (%) were converted using IFCC = (NGSP - 2.15) × 10.929. Given the pilot sample size and descriptive intent for glycemic endpoints, no p-values are reported for glycemic control.

Statistical processing of the results was performed using IBM SPSS Statistics (Version 21; IBM, Armonk, NY, USA). Graphs were performed using GraphPad (Prism version 8.42 (464) for MacOS, GraphPad Software, La Jolla California USA, www.graphpad.com).

## Results

### Subject demographics

12 patients were selected for inclusion in the study between October 2014 and March 2015. Two patients were excluded prior to the intervention due to the detected comorbidities. Ten patients (6 men and 4 women) were eventually enrolled in the study. The study population was 60 % male, had a mean age of 48 years (range: 22–58 years) and a mean baseline BMI of 41 kg/m^2^ (range: 34.7–46.2). Four subjects had type 2 diabetes, three were prediabetic (HbA1c>5.7 % and fasting glucose >100 mg/dl), and three were nondiabetic. Of the four diabetic subjects, three were receiving oral medications and one was treated with diet alone.

### Clinical outcomes

A progressive reduction in weight was observed in the first year following surgery. In the subsequent biennium, the weight demonstrated stability, with no substantial re-increase observed. The most significant weight reductions were documented during the initial six-month period following the procedure. In the subsequent follow-up period, a slow and stable weight loss was observed. The mean Excess Weight Loss at 36 months was 43.2 %. Subjects’ anthropometric changes are summarized in Table 1.

### Glycemic control

In patients with diabetes (n=4), the median fasting blood glucose level was 7.42 mmol/l [6.29–13.39]; 95 % CI 5.83–18.69 at baseline. After a period of 36 months (n=4), the median value was found to be 6.63 mmol/l [6.14–8.0]; 95 % CI 6.02–9.0. In the prediabetic subjects (n=3), the median fasting blood glucose level at the commencement of the study was 6.56 mmol/l [6.31–6.68], with a 95 % confidence interval of 6.06–6.8. Following a 36-month period (n=2), the median value was determined to be 5.5 mmol/l [5.5–5.5], with a 95 % confidence interval of 5.44–5.55. In non-diabetic subjects (n=3), the initial fasting blood glucose levels were recorded at 5.96 mmol/l (n=3), with a range of 5.84–6.22 mmol/l and a 95 % confidence interval of 5.72–6.49 mmol/l. After a period of 36 months (n=3), the median value was determined to be 5.08 mmol/l [4.61–5.08]; 95 % CI 4.13–5.09.

The HbA1c value in diabetics (n=4) at the commencement of the study demonstrated a median of 48.0 mmol/mol [41.5–76.5]; 95 % CI 39.0–101.0. Following a 36-month period (n=4), the median value was determined to be 38.0 mmol/mol [32.5–48.5]; 95 % CI 32.0–54.0. The median HbA1c value in subjects with prediabetes (n=3) at the commencement of the study was 40.0 mmol/mol [39.0–47.5]; 95 % CI 38.0–55.0. After a period of 36 months (n=2), the median value was determined to be 34.0 mmol/mol [31.0–35.5]; 95 % CI 28.0–37.0.

The HbA1c value in non-diabetic subjects (n=3) at the commencement of the study was 35.99 mmol/mol [35.5–41.0]; 95 % CI 35.0–46.0. After a period of 36 months (n=3), the median value was found to be 28.96 [28.48–30.98]; 95 % CI 28.0–33.0.

**Fig. 2:**
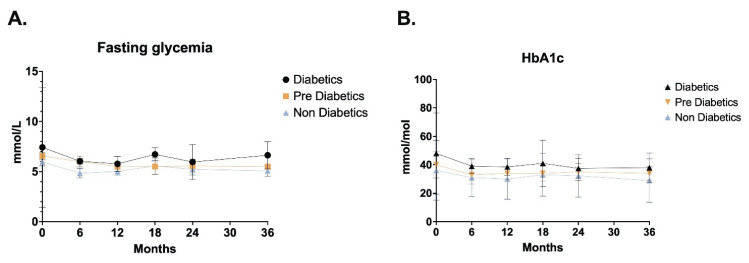
Changes of fasting glycemia and HbA1c from baseline through Month 36.

The changes of fasting glycemia and HbA1c are shown in Figure 2.

### Preoperative and postoperative micro- and macronutritional parameters

In the week before the operation, venous blood was taken to evaluate possible preoperative deficiencies of micro- and macronutrients. At least one deficiency was found in 80 % of the patients enrolled in the study. An overview of the incidence of nutritional deficiencies before and after surgery is shown in Table 2.

Plasma concentrations of both vitamin B1 and B2 changed statistically significantly. Vitamin B1 levels increased gradually throughout the period in all subjects. Plasma B2 levels decreased significantly over 36 months. But none of the patients had deficient levels of B1 or B2 vitamin preoperatively and in the follow-up period after surgery. In terms of fat-soluble vitamins, we have seen fluctuations, especially vitamin D3. Here, due to seasonal effects, there was an increase and then a decline. However, at 12 months, at the same time of the year, the level of vitamin D3 after surgery was significantly higher compared to baseline. Similar fluctuations in levels as in vitamin D3 were reported for 25 (OH)D.

There were no significant changes in blood levels of vitamin A. In the case of vitamin E, no deficiencies were noted, however, a significant decrease in levels occurred during the observation period, but not below the physiological range. One patient had deficiency of vitamin B12 before PJD. During the follow-up period, vitamin B12 blood levels gradually decreased in all patients. 8 patients had vitamin B12 deficiency 36 months after surgery. Folate blood levels increased slightly during the follow-up period after surgery and no patient was deficient. Iron blood levels increased slightly, especially in the first half of the follow-up period. 1–2 patients had iron deficiency during the study, and all patients had normal blood iron levels at the end of the study. The results of the preoperative and postoperative nutritional profile are presented in Table 3.

### Adverse events after PJD

The most common complications arising from PJD were diarrhea, flatulence and abdominal pain in the trocar region. Postoperatively, four patients exhibited flatulence, eight experienced diarrhea, and two reported abdominal pain. Over the course of a three-year period, this condition demonstrated signs of improvement in a number of patients. Following a dietary intervention aimed at reducing fat intake, a positive response was observed in terms of improvements in diarrhea and flatulence. Three years following the administration of PJD, two patients reported flatulence, while four reported diarrhea. One patient reported the occurrence of abdominal spasms 12–36 months after PJD. One patient experienced a serious complication of ileus 22 months after PJD. In one patient, the anastomosis was reversed at the patient’s request 31 months after surgery.

## Discussion

In this longitudinal study, the effect of intestinal anastomosis on glycaemic control, incretin regulation and weight reduction were evaluated. All patients enrolled in the study experienced weight reduction throughout the 36-month follow-up. The highest weight loss we recorded in the first year of follow-up, in the following years was a slight but permanent decrease. We did not notice any increased weight gain in any of the patients. The mean weight loss in all patients was 19.1 kg with an EWL of 43.9 %. Weight loss is comparable to similar endoscopic procedures [11]. Weight loss is then compared with the results of a study by Melissas, who performed a similar intestinal diversion in 6 patients [12]. In contrast to our patients, where we observed a steady decrease in weight even over a period of 36 months, in his study lasting the same period, there was a gradual increase in weight.

Weight reduction in obese individuals is commonly accompanied by favorable metabolic changes, including improved fasting glucose and HbA1c levels [13]. In this study, gradual improvements in glycemic markers were observed across all subgroups, consistent with enhanced metabolic flexibility secondary to weight loss [14,15].

These effects align with previously reported metabolic benefits of bariatric [16] and endoscopic interventions [17], though the current procedure was primarily designed to achieve durable weight reduction with a minimal risk of nutritional deficiency rather than to treat diabetes specifically [18].

Limitations of our study include the relatively small number of subjects enrolled, all of whom were recruited from a single center, and the fact that there is no control group.

Preoperative serum levels of the vitamins A, E, B1, B2, B6, folate, ferritin, total protein and albumin were in normal range in all patients. A few patients had deficit of vitamin B12 (10 %) and iron (10 %), prealbumin (10 %) and creatinine (10 %). The serum values all of these parameters were in normal range on average in the whole group of the patients.

But almost all patients had low serum levels of vitamin D3 and 25(OH)D before surgery (90 % and 100 %). Vitamin D deficiency is very common in morbidly obese patients. An inverse relationship between BMI/total body fat and serum 25(OH)D concentrations has been reported in several studies [19–21].

Given that the duodenum, jejunum, and ileum are involved in nutrient absorption, bariatric surgery could induce intestinal malabsorption of the vitamin B complex (vitamin B1, 2, 3, 5, 6, 7, 9, 12), fat soluble vitamins, minerals and also macronutrients, mainly protein [22,23].

In this study, no vitamin A, E, B1, B2, B6, folate or ferritin or albumin deficiency was detected in any of the examined subjects during three years after surgery. These results are favorable in comparison with the results of the other malabsorptive surgeries which report 10–11 % of vitamin A deficiency in the first year following RYGB and biliopancreatic diversion (BPD) [24], prevalence of vitamin E deficiency 4.8 % in the first year following BPD [24,25], vitamin B1 deficiency 30 % within 4–6 weeks after surgery [26], an average of 38 % of patients with folate deficiency which progresses asymptomatically after various types of bariatric surgery and 13 % patients with folate deficiency in the first year after RYGB [27]. 1 patient has protein deficiency 1 and 2 years after surgery, but there was no patient with protein deficiency in this study 3 years after surgery.

Two patients had iron deficiency in the first year after surgery, which represents a 10 % increase. After 2 years, 1 patient had iron deficiency. Three years after the operation, there was no iron deficiency in the subjects of the studied group. The prevalence of iron deficiency and iron-deficiency anemia ranges from 20 to 70 % after RYGB [28].

The lower occurrence of deficiencies of various vitamins and protein after the jejuno-ileal intestinal diversion is probably due to the fact that after this operation only part of the chyme proceeds through the anastomosis and the native path remains open in contrast to malabsorptive procedures such as RYGB or BPD with duodenal switch [29]. Moreover, the size of the stomach is preserved, and patients report fewer digestive problems after this operation unlike malabsorptive procedures with stomach size reduction. The stomach change usually causes a low tolerance for red meat, reduced acidity in the stomach and essential chronic use of drugs to suppress the secretion of gastric acid [22].

A statistically significant gradual decrease in the concentration of vitamin B12 in the blood was detected. There was a gradual increase in the incidence of B12 deficiency from 10 % before surgery to 20 % in the 2^nd^ year and 80 % in the 3^rd^ year after surgery.

The complexity of vitamin B12 absorption makes vitamin B12 deficiency very common in any clinical situation involving the gastrointestinal tract, including bariatric surgery. Various studies of malabsorptive and mixed techniques report from 6.5 % to 75 % causes of vitamin B12 deficiency from 12 month to 5 years after surgery. As time passes after bariatric surgery, the body’s stores of this vitamin are depleted, so prophylactic supplementation is recommended [34]. The clinical symptoms like anemia and neurological symptoms develop after approximately 2–3 years of insufficient intake of vitamin B12 [30,31].

There were significantly higher concentrations of vitamin D3 and 25(OH)D in blood 12 months after surgery. The occurrence of vitamin D3 and 25(OH)D deficiency was reduced by 50 % in the 1^st^ year after surgery, but in subsequent years the concentrations were similar to values before the operation. In the 3^rd^ year after the operation the occurrence of the vitamin D3 and 25(OH)D deficiency was 10 % lower than before the operation. Vitamin D status depends on a number of factors such as skin exposure to UV-B radiation and diet. Bariatric surgery, especially when malabsorptive techniques are used, favors vitamin D deficiency, which is observed in more than 70 % of cases [28].

But on the other hand, some of the authors report significantly increased serum 25(OH)D after weight reduction and after reduction in the volume of visceral adipose tissue (assessed by computed tomography) [32,33].

## Limitations of the study

The study design and sample size, as well as the duration and completeness of the follow-up, are all crucial factors in the analysis.

This was a single-arm pilot study with a small sample size (n=10) and no control group, which limits the generalizability of the results and precludes causal inference. The 36-month follow-up, while informative, remains relatively brief for bariatric and metabolic endoscopy, where ≥4–5-year outcomes and long-term safety surveillance are increasingly reported. The implementation of an extended follow-up, accompanied by a systematic capture of adverse events and retention, would serve to enhance confidence in the durability and safety of the intervention.

The clinical interpretation and external comparability of the data must be considered.

The cohort comprised patients with type 2 diabetes, prediabetes, and no diabetes. It should be noted that the study was not designed to provide robust subgroup analyses. The absence of a comparator (e.g., surgical procedures, endoscopic sleeve gastroplasty, or a duodenal-jejunal bypass liner) limits contextualization of effect sizes. Furthermore, changes in dietary patterns and health-related quality of life – key determinants of real-world adoption – were not systematically assessed. It is recommended that future studies incorporate validated measures of diet and quality of life, in addition to a comparative design. The incorporation of these measures will facilitate clinical translation.

## Conclusions

Durable weight loss and glycemic improvement at 36 months in a first-in-human endoscopic dual-path diversion cohort.

In this single-arm pilot, partial jejunal diversion created with an incisionless magnetic anastomosis system (IMAS) produced sustained weight loss and improvements in fasting glucose and HbA1c through 36 months, extending the one-year benefits reported in our original first-in-human study. Compared with small-intestine-targeted endoscopic therapies such as the duodenal-jejunal bypass liner (DJBL) – where most evidence concentrates at ~12 months – our 3-year data add durability signals in the absence of an explant schedule inherent to DJBL devices. These findings warrant confirmation in larger, controlled cohorts with ≥5-year follow-up.

Comprehensive micronutrient profiling suggests a favorable nutritional risk profile at 36 months.

A complete protein, vitamin and micronutrient panel revealed no clinically significant long-term deficiencies (except vitamin B12 and 25(OH)D) in this cohort, which contrasts with the high prevalence of late deficiencies consistently reported after bariatric surgery (e.g., total protein, albumin, prealbumin and vitamin B1, B2, B6, A, E, at ≤5 years) and aligns with the lower deficiency rates generally observed after stomach-targeted endoscopic sleeve gastroplasty at 12 months.

Significant decrease in blood vitamin B12 levels during the three years after surgery and persisted preoperative 25(OH)D deficiency at 24 and 36 months after surgery is comparable with the results of other malabsorptive techniques and requires long-term monitoring and supplementation of these vitamins.

Our 36-month, small-bowel-targeted endoscopic data therefore complement and extend existing endobariatric nutrition evidence beyond the first postoperative year.

Clinical translation will require standardized safety surveillance, diet/behavior assessment, and comparative effectiveness.

While the 3-year results support technical feasibility and metabolic benefit signals, future work should incorporate systematic adverse-event reporting and validated measures of dietary behavior and health-related quality of life, and should benchmark outcomes against established comparators (e.g., ESG, DJBL, metabolic surgery) to inform real-world adoption and shared decision-making. Recent DJBL syntheses and guidance underscore the value of comparative designs and standardized outcome frameworks that our next-phase studies will adopt.

## Figures and Tables

**Table 1:** Overview of patient weight reduction from baseline through Month 36.

	Baseline	6 months	12 months	18 months	24 months	36 months
*Weight [kg]*	120.9 ± 17.8	108.0 ± 16.3	103.7 ± 21.5†	104.2 ± 23.4†	103.1 ± 22.9†	101.9 ± 22.2†
*BMI [kg/m* *^2^* *]*	41.28 ± 4.4	36.9 ± 4.7†	35.4 ± 6.6†	35.6 ± 7.5†	35.2 ± 7.4†	34.8 ± 7.2†
*kg lost [kg]*	---	12.9 ± 7.7	17.3 ± 11.9†	16.8 ± 14.9	17.8 ± 15.1	19.1 ± 15.6
*TWL [%]*	---	10.6 ± 5.9	14.6 ± 11.4	14.2 ± 13.7	15.0 ± 13.4	15.9 ± 13.7
*EWL [%]*	---	28.3 ± 17.3	40.2 ± 36.7	39.1 ± 42.4	41.9 ± 41.3	43.2 ± 41.8

The data are expressed as the mean ± standard deviation. TWL – Total Weight Loss; EWL – Excess Weight Loss;

† Statistically significant difference vs. baseline (*p*≤0.05); comparison performed by Wilcoxon test.

**Table 2:** Nutritional deficiencies before and after surgery (n=10).

*Nutrients [deficiency/units]*	Baseline	Patient with deficiency [n]		36 months
		12 months	24 months	
*Vitamin A [<1.05 μmol/l]*	0	0	0	0
*Vitamin E [<12 μmol/l]*	0	0	0	0
*25(OH)D [<50 nmol/l]*	10	5	9	9
*Vitamin D3 [<50 nmol/l]*	9	4	7	8
*Vitamin B1 [<65 nmol/l]*	0	0	0	0
*Vitamin B2 [<100 nmol/l]*	0	0	0	0
*Vitamin B6 [<12.5 nmol/l]*	0	0	0	0
*Vitamin B12 [<148 pmol/l]*	1	1	2	8
*Folate [<3.1 μg/l]*	0	0	0	0
*Iron [<12.5 μmol/l]*	1	2	1	0
*Ferritin [<15 μg/l]*	0	0	0	0
*Prealbumin [<0.2 g/l]*	1	1	1	1
*Albumin [<35 g/l]*	0	0	0	0
*Total protein [<64 g/l]*	0	1	1	0
*Creatinine [<49 μmol/l]*	1	1	1	1

**Table 3:** Preoperative and postoperative nutritional profile of patients (n=10).

	Baseline	12 months	*p*	24 months	*p*	36 months	*p*
*Vitamin B1 [nmol/l]*	124.7 ± 21.2	126.4 ± 21.8	0.889	173.4 ± 37.1	0.012	172.6 ± 27.9	0.012
*Vitamin B2 [nmol/l]*	375.8 ± 63.3	370.6 ± 46.3	0.575	295.2 ± 37.7	0.012	311.0 ± 26.0	0.017
*Vitamin B6 [nmol/l]*	44.9 ± 28.9	59.7 ± 52.3	0.878	68.1 ± 74.6	0.074	48.2 ± 20.4	0.646
*Vitamin B12 [pmol/l]*	258.3 ± 96.4	218.4 ± 53.9	0.241	165.2 ± 38.5	0.028	112.4 ± 36.3	0.007
*Folate [μg/l]*	10.4 ± 6.3	15.2 ± 5.7	0.114	11.1 ± 4.3	0.959	11.0 ± 2.7	0.646
*Vitamin A [μmol/l]*	2.3 ± 0.6	2.3 ± 0.7	0.507	2.2 ± 0.7	0.445	2.5 ± 0.9	0.878
*Vitamin E [μmol/l]*	32.9 ± 4.8	23.3 ± 5.1	0.013	23.1 ± 4.9	0.007	24.5 ± 2.7	0.017
*Vitamin D3 [nmol/l]*	34.7 ± 14.4	70.4 ± 28.0	0.013	41.9 ± 18.7	0.285	38.0 ± 15.3	0.646
*25(OH)D [nmol/l]*	23.4 ± 11.1	47.9 ± 17.6	0.009	28.1 ± 13.7	0.575	30.8 ± 10.4	0.169
*Iron [μmol/l]*	17.6 ± 6.3	21.6 ± 11.7	0.241	18.1 ± 4.7	0.646	19.2 ± 5.5	0.386
*Ferritin [μg/l]*	295.2 ± 226.9	198.5 ± 150.7	0.047	186.7 ± 146.7	0.028	153.9 ± 117.4	0.013
*Prealbumin [g/l]*	0.3 ± 0.1	0.3 ± 0.1	0.105	0.3 ± 0.1	0.313	0.3 ± 0.1	0.090
*Albumin [g/l]*	43.7 ± 3.4	43.3 ± 2.3	0.722	43.2 ± 2.1	0.574	43.6 ± 2.1	0.959
*Creatinine [μmol/l]*	74.8 ± 20.1	74.2 ± 20.1	0.722	76.1 ± 24.8	0.779	79.9 ± 30.1	0.646
*Total Protein [g/l]*	72.3 ± 4.0	69.5 ± 4.9	0.017	69.7 ± 4.1	0.053	70.2 ± 4.8	0.092
*Urea [mmol/l]*	4.6 ± 1.4	4.9 ± 1.7	0.201	5.3 ± 1.9	0.033	5.2 ± 1.9	0.066
*ALT [μkat/l]*	0.7 ± 0.2	0.5 ± 0.2	0.046	0.7 ± 0.2	0.878	0.5 ± 0.2	0.053
*AST [μkat/l]*	0.5 ± 0.1	0.4 ± 0.1	0.169	0.5 ± 0.2	0.575	0.5 ± 0.2	0.683
*ALP [μkat/l]*	1.3 ± 0.3	1.3 ± 0.3	0.386	1.4 ± 0.4	0.114	1.3 ± 0.5	0.959

The data are expressed as the mean ± standard deviation. *p* – comparison difference vs. baseline performed by Wilcoxon test.
